# Phage Peptide Libraries As a Source of Targeted Ligands

**Published:** 2016

**Authors:** A. A. Nemudraya, V. A. Richter, E. V. Kuligina

**Affiliations:** Institute of Chemical Biology and Fundamental Medicine, Siberian Branch of the Russian Academy of Sciences, Lavrentiev Ave., 8, 630090, Novosibirsk, Russia

**Keywords:** targeted peptides, drug delivery, phage display, phage peptide libraries

## Abstract

One of the dominant trends in modern pharmacology is the creation of drugs that act directly on the lesion focus and have minimal toxicity on healthy tissues and organs. This problem is particularly acute in relation to oncologic diseases. Short tissue- and organ-specific peptides capable of delivering drugs to the affected organ or tissue are considered promising targeted agents that can be used in the diagnosis and therapy of diseases, including cancer. The review discusses in detail the technology of phage display as a method for obtaining specific targeted peptide agents and offers examples of their use in diagnostic and clinical practice.

## INTRODUCTION

Drug delivery directly to the lesion focus is one of the main challenges in modern pharmacology. This problem is particularly acute in relation to oncologic diseases. Generally, anticancer drugs exhibit significant toxicity, affecting healthy cells and tissues alongside malignant ones. Therefore, the development of principally new antitumor agents, the effectiveness of which is provided by a selective effect on the tumor, is considered as one of the key areas in antitumor therapy. The appearance of such tumor-targeted drugs would allow us to reduce the effective therapeutic dose and minimize side effects.

Cancer cells are known to have many quantitative and/or qualitative characteristics that distinguish them from normal cells. For instance, the expression of growth factor receptors, such as epidermal growth factor receptors (EGFR), transferrin or folate receptors, is often higher in tumor cells, which allows for their uncontrolled proliferation and promotes metastatic processes [1]. Tumor growth is also known to be accompanied by active processes of angiogenesis, which are mainly activated in an adult organism during the regeneration of damaged tissues. The processes of angiogenesis can be activated, for example, upon overexpression of vascular endothelial growth factors (VEGF) [2]. Finally, there are physical differences between tumor and normal tissues: temperature change, low oxygen concentration (hypoxia), and reduced pH [3].

The unique properties of cancer cells allow one to find specific ligands that interact directly with the tumor and to conduct targeted therapy of malignant tumors.

Phage display technology is one of the promising approaches in the search for tissue- and/or organ-specific molecules. Combinatorial phage peptide libraries allow one to obtain highly specific peptides, including peptides specific to various types of tumors. The search for tumor-specific peptides using combinatorial phage peptide libraries can be carried out *in vitro *and *in vivo*. Currently, such tumor-specific peptides are considered as targeting vehicles for the delivery of therapeutic genes, cytokines, agents for imaging, proapoptotic peptides, and cytotoxic drugs.

This article reviews in detail phage display technology as a method for obtaining a targeted agent capable of ensuring specificity of interaction between a drug and the target organ or tissue. Examples of the use of organ- and tissue-specific peptides in biomedicine are given.

## PHAGE DISPLAY TECHNOLOGY

**Fig. 1:**
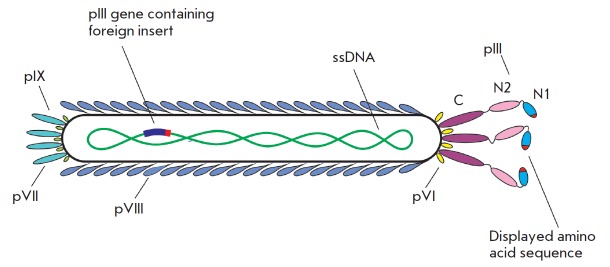
The structure of a bacteriophage with a displayed foreign amino acid sequence in a pIII surface protein (indicated in red). N1, N2 and C – domains of the pIII surface protein

Phage display technology, first proposed by G.P. Smith in 1985, played an important role in the development of fundamentally new approaches in molecular biology and opened up new opportunities for the development of the pharmaceutical industry. The concept of phage display lies in the cloning of a foreign DNA sequence into a specific site of a bacteriophage surface protein gene so that this sequence shares the reading frame with the protein. The result is a chimeric protein containing a foreign amino acid sequence formed (displayed) on the surface of the bacteriophage (*Fig. 1*). Furthermore, the physiological properties and viability of the viral particle are preserved [4, 5].

The technology of phage display has been developed for various bacteriophages; for example, λ, T4 and T7 [6-8]. The most widely used in phage display construction are filamentous bacteriophages [9], the virions of which resemble a long, thin thread. Filamentous phages are small and have a simply arranged genome [10]. The most studied filamentous phages – M13, f1, and fd – are under the genus *Inovirus*, family *Inoviridae* and combined into the Ff group since they infect *Escherichia coli *carrying F-pili [11]. Ff strain phages contain circular single-stranded DNA, which is 98.5% identical among various strains in the group [10]. The genome of Ff phages consists of 11 genes, the products of which can be grouped according to their functional purpose: capsid proteins – pIII, pVI, pVII, pVIII, pIX; proteins involved in DNA replication – pII, pV, pX; and proteins responsible for the assembly of phage particle – pI, pIV, pXI (*Fig. 1*) [12].

Typically, filamentous phages infect Gram-negative bacteria (*Escherichia*, *Salmonella*, *Pseudomonas*, *Xanthomonas*,* Vibrio*, *Thermus *and *Neisseria*). A bacterial cell infected with the phage releases new viral particles but does not undergo lysis.

Depending on which surface protein gene the foreign DNA is cloned into, there are several types of phage display (*Table*).

**Table:** Types of phage display depending on the surface protein used

Type of phage display	Used surface protein (whether all copies of the protein represent foreign sequence)	Number and localization of surface protein gene copies
3	pIII (all)	1 in bacteriophage genome
8	pVIII (all)	1 in bacteriophage genome
33	pIII (partially)	2 in bacteriophage genome
88	pVIII (partially)	2 in bacteriophage genome
3+3	pIII (partially)	2 in bacteriophage genome and phagemid vector
8+8	pVIII (partially)	2 in bacteriophage genome and phagemid vector

The proteins pIII and pVIII (406 and 50 amino acid residues, respectively), which are also called the minor and major proteins, are the most applied in the technology of phage display for the introduction of a foreign amino acid sequence. Both proteins have N-terminal signaling sequences, which are cleaved by a signal peptidase during protein maturation after transfer to the internal side of the bacterial membrane. Mature proteins incorporate into the phage envelope during its assembly. Thus, in order for the foreign peptide to be displayed on the phage particle surface, its encoding nucleotide sequence should be cloned between the sequence of the surface protein and signaling sequence in the same translation frame [13].

Bacteriophage contains three to five copies of the pIII protein. Together with pVI, they form a distal cover of the virion and are necessary for its stabilization and the termination of phage particle assembly during the release from the bacterial cell. Moreover, pIII plays an important role in infection by attaching to the bacterial cell via F-pili [14]. Protein pIII contains three domains: N1, N2, and C separated by glycine spacers (*Fig. 1*). Domain C is responsible for virion assembly, while N1 and N2 are required for the infection of bacterial cells [15]. If a short nucleotide sequence is embedded in the *pIII *gene, the foreign insert will be carried by every molecule of the pIII protein. The phage display in this case is called type 3 display (*Table*).

One phage particle contains about 2,700 copies of the pVIII protein that forms the bacteriophage envelope and has a spiral structure. Four positively charged lysine residues, which interact with the negatively charged phosphate groups of viral ssDNA inside the phage, are located at the C-terminus of the protein. Ntermini are located on the outside of the viral particle [16, 17]. The maximum length of the foreign insert that does not lead to significant aberrations of the phage particle assembly and is displayed on each pVIII protein is 6–7 amino acid residues (type 8 phage display) [18, 19].

The loss of chimeric protein function takes place upon display of long heterologous amino acid sequences, which should be replenished with wild-type pIII or the pVIII protein. There are systems with a phage genome containing *pIII *(*pVIII*) genes of two types: recombinant and wild. As a result, only a portion of pIII (pVIII) proteins carries heterologous sequences, while the other part preserves native functions (type 33 (88) phage display) [20]. Replenishment of the lost function of the protein can occur in systems using phagemid vectors and helper phages [21, 22]. The Phagemid vector in such a case contains a plasmid and phage origins of the replication, the sequence encoding an antibiotic resistance gene, and the sequence encoding the chimeric protein. The helper phage encodes a wild-type protein necessary for the proper assembly of viral particles. Upon infection, the wild-type gene enters the *E. coli *cell, along with the helper phage, with the recombinant gene in the plasmid. As a result, mature particles of the released bacteriophage are arranged in a mosaic pattern; i.e., they contain wild-type and recombinant proteins (type 3+3 or 8+8 phage display) [20].

The first studies of phage display technology application were aimed at obtaining peptides and proteins capable of specifically binding to antibodies. In his pilot work, Smith G.P. obtained a phage clone (fECO1) that contained the pIII protein with an inserted fragment of EcoRI restrictase. This clone was effectively neutralized by antibodies against restrictase [4]. Further development of this work yielded numerous other experiments in which phage particles with a displayed antigen served as immunogens capable of eliciting an immune response [23-25].

In the second part of his work, Smith investigated the possibility of enriching a mixed population of bacteriophages with specific fECO1 phages by affinity binding with antibodies against EcoRI. A mixture of phage fECO1 and a considerable excess in the wild-type M13mp8 phage were added to the absorbed antibodies against EcoRI. Unbound phages were washed away with the medium, while absorbed phages were eluted with an acidic buffer, neutralized, and titrated. As a result of three consecutive experiments, a population enriched with fECO1 phage 1,500–7,200 times more than in the case of the wild-type phage was obtained [4]. At this stage, an idea appeared of using antibodies for the selection of specific clones from a population of bacteriophages (combinatorial phage library) where each individual phage particle displays a random amino acid sequence on its surface. Since 1988, the procedure of affinity enrichment of a phage population with a specific bacteriophage has become known as biopanning [26].

**Fig. 2:**
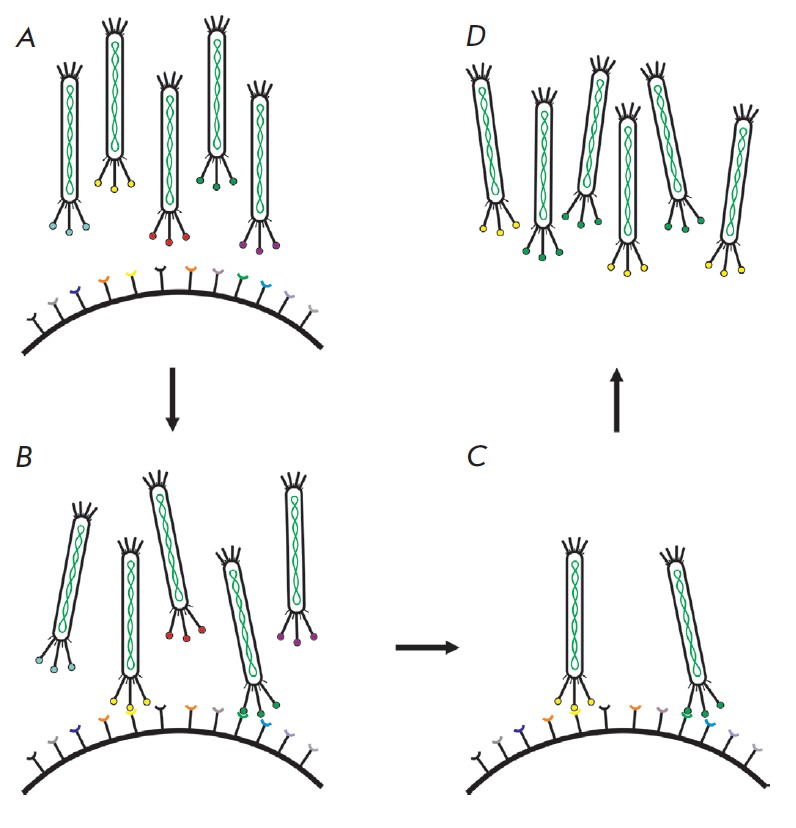
Schematic representation of a typical biopanning of a phage library. *A, B *– incubation of a combinatorial phage library with a target; *C *– washing-off of the unbound phages; *D *– elution of bound phages and their amplification for the next rounds

A typical biopanning round comprises the following steps: 1) incubation of a combinatorial phage library with the target (protein, cell culture, tumor tissue, etc); 2) washing-off of unbound phages; 3) elution of bound phages; and 4) amplification of the eluted phage for the next rounds (*Fig. 2*).

After several rounds of biopanning, the rate of population enrichment with the bacteriophage is determined by titration and/or immunoenzyme techniques. Then, individual phage clones are isolated and the sequence of the foreign insert is determined. It is important to note that a simple physical bond between the displayed peptide and a sequence cloned into the phage genome allows for an easy analysis of the insert primary structure. Due to the small size of filamentous bacteriophages (5 nm diameter, 1 μm length), the concentration of phage particles can be as high as 10^14^ particles/ ml, which allows for the screening of a large number of variants. The representativeness of the peptide phage library reaches 10^9^ different insertion variants [27].

Nowadays, combinatorial phage libraries are widely used as a tool that allows one to solve various tasks in molecular biology, biochemistry, and biomedicine. Libraries can be performed based on random combinations of oligopeptides, antibodies, enzymes, fragments of genomic DNA, cDNA, open reading frames, or other functional genomic regions [28-30]. Library screening allows one to select molecules with specific properties, study protein-protein interactions, investigate markers of specific tissues, organs and biochemical processes, search for the substrates of various enzymes, epitopes of antigens and paratopes of antibodies and, finally, obtain highly specific molecules with the desired properties [31, 32].

## SELECTION OF SPECIFIC PEPTIDES FROM PHAGE LIBRARIES

Construction of a peptide library is one of the key moments in successful screening, since the probability of a ligand selection that specifically binds to a certain target depends considerably on the library diversity and insert length. One of the most common strategies for constructing a combinatorial library of peptides is based on the triplet rule and degeneracy of the genetic code. The strategy is to generate various combinations based on a (NNK)_n_ codon, where N is the equimolar ratio of all four nucleotides, and K stands for the mixture of guanine and thymine only (1 : 1). Due to the use of the codon (NNK)_n_ instead of (NNN)_n_, the number of possible stop codons is reduced from three (TAA, TGA, TAG) to one (TAG) and the probability of coding different amino acids is aligned. Thus, 32 possible (NNK)_n_ codon variants encode 20 canonical amino acids and one stop codon [13]. The number of possible variants of amino acid sequence of length *n *is equal to 20^n^. However, other factors such as the presence of a stop codon in the peptide sequence and transformation efficiency of* E. coli *cells with phage constructs affect the representativeness of the library in practice. Typically, the representativeness of a commercial phage peptide library is about 10^9^ phage particles [33]. Furthermore, the peptide insertion may be both linear and circular due to the formation of disulfide bridges between the cysteine residues flanking the insert.

As mentioned above, a heterologous insert can be displayed either by the pIII or pVIII protein. Libraries based on pIII, which is represented by only three to five copies at one of the ends of the viral particle, are used for the generation of highly specific ligands with high affinity for the target. Ligands with a dissociation constant in the range of 1–10 μM are obtained using such libraries [34]. Such specific peptides are most commonly used for the delivery of various substances to a target or imaging of specific structures and biochemical processes.

The major protein pVIII covering the capsid provides multivalent binding and high avidity, which adversely affects the affinity of interaction between peptide and target. Using pVIII-protein-based libraries, ligands with lower individual affinity are selected; the dissociation constants of such ligands are in the range of 10–100 μM [34]. However, these libraries are also widely used since the selected phage particles show a high affinity for the target, stability and they can be easily produced in large amounts. For example, Lang Q. et al. applied enzyme immunoassay in the detection of a prostate-specific antigen (PSA) using a phage clone selected from the pVIII library [35]. The possibility of targeted delivery of GAPDH siRNA to cancer cells using the ability of phage proteins to self-assemble in the presence of any nucleic acid is shown [36]. A phage peptide library based on the pVIII major protein was used for the selection of clones specific to human breast adenocarcinoma cells MCF-7. Recombinant pVIII proteins of this clone were generated by conventional amplification of the phage clone separated from the phage particle and incubated with GAPDH siRNA to form so-called nanophages. The resulting particles (nanophages) protected GAPDH siRNA from degradation by plasma nucleases, provided their specific delivery to MCF-7 cells, and internalization into the cell but did not affect the functionality of GAPDH siRNA.

Screening of phage peptide libraries can be carried out both *in vitro *and *in vivo*. *In vitro *screening is conducted using a variety of objects: inactivated viruses and bacteria, purified protein fractions, enzymes, receptors, functional domains, and cell cultures [37, 38].* In vitro *screening also includes selection using inorganic molecules (e.g., metals) and synthetic materials [39].

The most simple and direct method of *in vitro *selection of specific peptides is the selection on a purified substance of the target protein. For example, using screening of a phage peptide library displaying a seven-residue peptide with a fibroblast growth factor 8 (FGF8b), a HSQAAVP (P12) peptide was obtained that specifically binds to the receptor of this factor. FGF8b is a major isoform that is produced by prostate cancer cells. The selected P12 peptide inhibits cell proliferation induced by FGF8b, causes cell cycle arrest in the G0/G1 phase by suppression of cyclin D1 and PCNA, and block activation of the Erk1/2 and Akt cascades in both prostate cancer cells and vascular endothelial cells. Thus, P12 acting as an antagonist of FGF8b is a potential therapeutic tool in prostate cancer [40].

The disadvantage, or rather limitation of such a selection method, is that the peptides obtained after screening on the substance of purified proteins may not have the targeted properties *in vivo*. One of the reasons for this can be the specific post-translational modification of proteins that occur during various processes, including malignant cell transformation. Moreover, obtaining a soluble substance of a purified protein while maintaining its native structure and function is not always a trivial task [41].

*In vitro *screening on cell cultures allows one to successfully select peptides to various cell surface structures and also peptides capable of internalization into cells both through receptor-mediated and non-receptor- mediated ways: so-called cell-penetrating peptides (CPP) peptides. The advantages of a selection on cell cultures include the ability to obtain peptides specific to a particular cell type without knowledge of the particular target the peptides bind to.

Phage peptide libraries with additional properties are constructed on the basis of the vast data obtained. Thus, for example, a new class of peptide phage libraries (iPhage libraries) exists in which the displayed peptide is able to internalize a phage particle into the cell and provide specific binding to the cellular organelles and functional domains of intracellular proteins. The peptides selected from such a library allow one to study intracellular signaling and metabolic pathways [42].

A less common means for screening a phage peptide library is different biological fluids. Thus, a large amount of fibrin is found in the extracellular matrix of a tumor, which is caused by a constant penetration of fibrinogen into the tumor stroma and its cleavage. Pilch J. *et al*. performed biopanning with coagulated plasma and selected two cyclic decapeptides (CLT1 and CLT2) capable of specifically binding to fibrinfibronectin complexes. Intravenous administration of fluorescently labeled CLT1 and CLT2 in mice with different grafted tumors led to their accumulation in the extracellular space of the tumor. The selected peptides also specifically bound to lesion sites in tissues. Thus, such peptides can be useful for the development of targeted drugs for the diagnosis and therapy of tumors and damaged tissues [43].

The possibility of selecting specific peptides by *in vivo *screening of a phage peptide library was shown by Pasqualini and Ruoslahti in 1996 [44]. There are several ways to introduce a phage peptide library into experimental animals in *in vivo *screening. The most common is intravenous administration, which allows almost immediate introduction of the library into blood vessel receptors, organs, and tissues. During circulation of a phage peptide library in the bloodstream, part of the phage population binds to plasma proteins and other non-target organs and tissues. This part of the library does not get involved in further rounds of selection, because only the associated-with-the-target-organ part of the library is amplified.

However, intravenous administration complicates the selection of peptides specific to brain structures, because penetration of phage particles is limited by the blood-brain barrier. Intranasal administration of a peptide phage library was developed for this purpose. It has been established that the bulk of the substance is absorbed into the blood upon intranasal administration, while a smaller part enters the brain directly from the neurons of the olfactory tract with the help of perineural transport in sensory nerves and spreads through brain structures through mechanisms not associated with the blood flow [45, 46]. Nevertheless, the intravenous way of administration of a phage peptide library allows one to select peptides specific to the blood-brain barrier only [47]. These peptides have the potential to translocate to the inside of the blood-brain barrier and provide delivery of associated molecules to the brain structures.

An alternative to the intravenous and intranasal administration methods is introduction directly into the target (orthotopic). For example, intraperitoneal administration of a phage peptide library in mice with gastric cancer allowed one to select peptides that specifically bind to gastric cancer metastasis [48]. Orthotopic administration allows one to introduce all possible target library variants into the target and reduces the probability of phage particle capture by other organs. On the other hand, target properties of the peptides selected orthotopically are significantly reduced upon intravenous administration.

Finally, there is a transdermal way of administration, which allows one to select peptides capable of penetrating through intact skin [49, 50].

The main limitations of *in vivo *screening are nonspecific distribution of phage particles in organs and tissues and half-life of the introduced phage. It is shown that phage accumulates in considerable amounts in the liver and spleen upon circulation in the organism. Maximum concentration of the wild-type M13 phage is observed in the blood 5 and 15 minutes after intravenous administration in mice with an intact immune system (line CF-1) and mice with immunodeficiency, respectively. Then, the concentration of phage particles in the bloodstream decreases rather rapidly. It is important to note that the concentration of phage particles in the spleen of mice with immunodeficiency is much lower than in healthy ones, which indicates the ; involvement of the immune system, particularly the reticuloendothelial system, in phage capture [51]. The half-life of the wild-type M13 phage in a mouse bloodstream is about 4.5 hours, while various modifications of phage particles (e.g., glycosylation or succinylation) dramatically reduce the half-life (up to several minutes). Reduction of the half-life in the bloodstream and rapid degradation of modified phages are apparently associated with their interaction with the corresponding receptors and internalization in a cell [52]. These nuances must be considered in the construction and analysis of *in vivo *experiments.

In 2002, the results of the first screening of a phage peptide library carried out *in vivo *in a patient in coma were published [53]. After intravenous administration of such a library (one round of screening), biopsies of several organs were analyzed. It was shown that the distribution of 47,160 phage clones between organs was not coincidental. The experiment was the first step in the development of a molecular map of the distribution of human receptors. One of the selected phage-displayed peptides had a high affinity for prostate tissue and accumulated in it in considerable amounts. It was demonstrated later that this peptide is a ligand of interleukin- 11 [54].

Subsequently, phage particles selected from the biopsies of various organs after the first round of selection were combined into a new library. Two consecutive rounds of selection in two patients with prostate cancer were conducted using this library. A bioinformatic analysis of clones selected from various organs identified 15 peptides that could potentially serve as ligands of specific receptors. Bioinformatics methods (highthroughput analysis by similarity search, protein arrays) and affinity chromatography demonstrated that four of these 15 peptides are ligands of annexins A2 and A4, apolipoprotein E3, and leukocyte proteinase 3 [55].

Thus, one of the main advantages of using an *in vivo* system is that the targets for which the specific peptides are selected are presented in the natural microenvironment of the living organism.

## APPLICATION OF ORGAN- AND TISSUE-SPECIFIC PEPTIDES

Development of the technology for obtaining organand tissue-specific peptides using phage libraries and the discovery of new properties of these peptides has allowed researchers to consider them as promising diagnostic and therapeutic agents.

**Peptides with antitumor activity**

In most cases, the screening of phage peptide libraries is carried out in order to identify peptides that bind specifically to the receptor structures of the target organ or tissue and can subsequently serve as targeted agents for the delivery of different substances. On the other hand, organ- and tissue-specific peptides themselves have specific biological properties. In particular, some peptides exhibit antitumor activity.

For example, peptide LyP-1 that specifically binds to the lymphatic vessels of certain tumors inhibits the growth of human breast cancer MDA-MB-435 in model mice with severe combined immunodeficiency (SCID) upon regular intravenous administration. LyP-1 is shown to induce apoptosis of only the cells it binds to [56].

Cyclic peptide CIGB-300 blocks phosphorylation of serine-threonine protein kinase CK2, the synthesis of which is significantly elevated in various cancers. Impaired function of this enzyme leads to growth inhibition and induction of apoptosis of cancer cells in culture. CIGB-300 is also known to exhibit significant antitumor effect both upon local and systemic administration in mice with syngeneic tumors and human tumors and can serve as the basis for the development of anticancer drugs [57].

Peptide SMSIASPYIALE (peptide pIII) specific to GC9811-P endothelial cells of gastric cancer accumulating in the metastasis of this tumor was selected from a phage peptide library after four rounds of selection. A synthetic pIII analogue significantly inhibited the ability of GC9811-P cells for adhesion and invasion, impeded the development of metastasis and increased the lifespan of mice inoculated with a gastric cancer graft [58]. Afterwards, a GMBP1 peptide was obtained that specifically binds to the receptors of gastric cancer cells exhibiting multidrug resistance, and it contributed to cell phenotype alteration and restoration of drug sensitivity [59].

The acidic fibroblast growth factor (aFGF) is known to be produced by breast cancer cells and to promote tumor progression by interacting with the FGF receptor (FGFR). Peptide AP8 obtained from a phage peptide library is capable of specifically binding aFGF and inhibiting the proliferation of tumor cells and newly formed tumor vessels by arresting the cell cycle [60]. Such bifunctional peptides specific to tumor cells and tumor vascular cells can serve both as independent antitumor agents and vehicles for other drugs, enhancing their effect by their own anti-tumor action.

Wang H. *et al*. developed the strategy of joint application of the AVPI apoptotic peptide and DNA of the gene encoding the p53 protein for adjuvant therapy of breast cancer. The AVPI peptide was modified by addition of eight arginine residues. Due to the positively charged tail of arginine residues, AVPIR8 acquired the ability to effectively penetrate into cancer cells and serve as a vector for gene delivery due to the formation of nanocomplexes with the nucleic acid. Application of the AVPIR8/p53 DNA combination significantly increased the sensitivity of cancer cells to doxorubicin in experiments *in vitro*, as well as in breast cancer mouse models with a multidrug resistance phenotype [61].

A series of promising anticancer drugs was developed on the basis of tumor-targeted peptides. Several examples of anti-tumor peptides, which are at various stages of clinical trials (www.clinicaltrials.gov), can be noted. For example, a cyclic peptide [Arg-Gly-Asp- Dphe-(NMeVal)] containing an RGD motif serves as the basis for the Cilengitide antitumor agent. Cilengitide, a highly selective integrin inhibitor that arrests angiogenesis, is considered as the drug for central nervous system tumors, particularly glioblastoma, also small cell lung cancer, prostate cancer, and metastatic and/ or squamous cell carcinoma of the head and neck. Clinical trials of cilengitide (phase I/II) in combination with standard radiotherapy and temozolomide conducted in patients with newly diagnosed glioblastoma showed attainment of a primary endpoint (69% survival rate without progression for 6 months) [62, 63].

Clinical trials of the anticancer drug NGR-hTNF consisting of the human tumor necrosis factor (hTNF) and the NGR amino acid motif, the target of which is aminopeptidase N (CD13), began after the obtainment of encouraging results in antitumor therapy in animal models [64]. To date, there have been clinical trials conducted (phase I/II) for NGR-hTNF as a monotherapy drug for pleural mesothelioma and liver cancer. Clinical trials (phase I/II) of a combination of NGR-hTNF with such drugs as doxorubicin, oxaliplatin, capecitabine, gemcitabine, etc. in recurrent ovarian cancer, colorectal cancer, and small-cell lung cancer are at various stages of completion [5, 63]. According to the results of the clinical trials, NGR-hTNF is most effective in combination with conventional chemotherapy.

There are clinical trials (phase II/III) of oncolytic adenovirus Ad5-Δ24-RGD, a modified RGD capable of replicating in cells lacking a Rb/p16 signal pathway, as a drug for ovarian cancer and glioblastoma recurrence [5].

Encouraging results in completed and ongoing clinical trials inspire hope that soon there will be drugs based on tumor-specific peptides.

**Application of peptides in gene therapy**

Tumor-specific peptides are actively used as targeted components in the development of gene therapy drugs. For example, liposomes integrated into a membrane and peptides loaded with nucleic acid provide additional targeting of delivery structures. In the work by Yang Z. *et al., *two receptor-specific peptides were included in liposomes: angiopep and tLyP-1. Angiopep is specific to the receptor of low-density lipoproteins, the expression of which is enhanced in the blood-brain barrier structures. Peptide tLyP-1 is specific to the receptor of neuropilin-1, it effectively penetrates into tumor parenchyma. These modified liposomes loaded with siRNA suppressing gene expression of the vascular endothelial growth factor (VEGF siRNA) were efficiently transfected into U87MG human glioblastoma cells *in vitro *and reduced the expression of a target gene. The antitumor activity of the created modified liposomes was shown in a U87MG glioblastoma xenograft model* in vivo *[65].

A vector based on the adeno-associated virus (AAV), the capsid of which has an integrated peptide selected from the phage peptide library and containing an NGR-motif, was capable of targeted delivery of genetic information to CD13+ target cells. The CD13 receptor is expressed on endothelial cells of newly formed vessels and many cancer cells, which indicates that peptides containing a NGR-motif can be used as tumor-targeted agents [66].

Genetically modified bacteriophages displaying targeted peptides within one of their surface proteins can also be used as agents for targeted gene therapy. Among the important advantages of bacteriophages are their safety for humans, high stability of phage particles, and plasticity of the genome for construction [67, 68].

One of the first works that proved the possibility of targeted gene therapy with a modified bacteriophage was performed using a filamentous bacteriophage, a minor part of the pIII protein of which had an incorporated fibroblast growth factor (FGF2). The green fluorescent protein (GFP) gene under the cytomegalovirus (CMV) early promoter was used as a reporter gene. The modified bacteriophages specifically penetrated only into cells expressing the FGF2 receptor on the surface and internalized into the cell interior. Expression of the reporter gene and synthesis of GFP were observed [69]. Thus, bacteriophage, despite the lack of tropism for human cells, can be modified so that it acquires specificity to a particular cell type and the capacity to deliver foreign genetic material.

Bacteriophage M13 expressing the tumor-specific peptide RGD4C was used for the delivery of a transgene cassette regulated by the CMV promoter and flanked by AAV2 inverted terminal repeats. Phage particles were modified with cationic polymers in order to improve transfection properties. The modified phage particles possessed a higher antitumor activity compared to unmodified phages [70].

Finally, a tumor-specific peptide can be covalently attached to a therapeutic nucleic acid for its delivery to the target. For example, the possibility of delivery of VEGFR2 siRNA covalently linked to the targeted peptide cRGD was studied. Peptide cRGD specifically binds to αvβ3-receptors that are expressed at a high density on the endothelium of tumor vessels and in tumor cells. A covalent complex of cRGD-siRNA was shown to specifically enter αvβ3-positive HUVEC cells and turn off the target gene. The specific antitumor effect of the considered structures was identified in *in vivo *experiments in mice with immunodeficiency and inoculated A549 lung cancer tumor [71].

The obtained positive results allow one to consider tumor-specific peptides as a promising platform for the development of gene therapy agents.

**Targeted peptides in the diagnosis of diseases**

Peptides that specifically bind to certain tumor organs, tissues, cells, or vessels can be used for characterization of a cell culture, visualization of certain structures (including tumors) *in vivo, *and disease diagnostics [72].

For example, the RGD peptide conjugated with FITC is used in *in vitro *experiments to evaluate the expression level of αvβ3 integrins on the surface of various cancer cells in culture. Staining of human tumor biopsies embedded in paraffin using FITC-RGD allows one to evaluate the αvβ3 profile of the tumor tissue. This method of staining is much easier and cheaper than staining with antibodies to αvβ3 receptors [73].

Positron emission tomography (PET) is a radionuclide tomographic method of diagnostic study. The method is based on the detection of the distribution of compounds (radioligands) labeled with positron-emitting radioisotopes in an organism. Natural peptides (bombesin, somatostatin) are for the most part used as protein markers in PET [74]. Tumor-specific peptides can also be used as targeted agents for the delivery of radionuclide labels in the diagnosis of malignant neoplasms. Novel radioligands based on the RGD peptide are currently under clinical trials [75].

Another radioligand for PET imaging is based on a ^64^Cu-labeled NGR-containing peptide specific to the CD13 receptor. This compound bound to CD13^+^ HT- 1080 cells and showed no tropism for CD13^-^ MCF-7 cells in experiments *in vitro*. Results of *in vitro *experiments were confirmed *in vivo *using HT-1080 and MCF-7 tumor xenografts [76].

Tumor-specific peptides can conjugate not only with radionuclides, but also with other diagnostic agents, such as paramagnetic substances for MRT (magnetic resonance tomography), SPECT (single photon emission computed tomography), or fluorescent dyes in case of FOT (fluorescence optical imaging) [74]. These conjugates selectively accumulate in tumor at concentrations greatly exceeding their concentration in other organs, thereby amplifying the signal detected by the device.

Thus, tumor-specific peptides have significant potential for improving existing technologies of diagnostics and imaging of tumor structures.

**Peptides: agents for targeted drug delivery**

An example of using tumor-specific peptides for the delivery of pro-apoptotic proteins is a protein that combines the pro-apoptotic peptide KLAK and targeted peptide RGD. The RGD motif of the peptide recognizes integrin receptors, which are expressed in a large number on newly formed vessels and cancer cells [77]. The obtained bifunctional protein specifically binds to target cells (tumor endothelial cells), penetrates into the cells, and induces their apoptosis through the mitochondrial pathway [78].

Peptide M2pep, which specifically binds to tumorassociated macrophages and mouse M2 macrophages, was proposed as an agent for the targeted delivery of the KLA pro-apoptotic protein [79]. Tumor-associated macrophages play an important role in tumor progression by stimulating tumor cell growth, angiogenesis and metastasis, and promote drug resistance [80]. The resulting recombinant protein M2pepKLA inhibited tumor growth and reduced the population of tumorassociated macrophages [79].

Peptide C**RGDK**GPDC (iRGD) combining the properties of two motives, RGD (integrin-binding) and R/ KXXR/K (neuropilin (NRP)-binding), was selected based on the T7 phage peptide using a phage peptide library [81, 82]. RGD guides the peptide to the tumor, while R/KXXR/K increases the permeability of tumor vessels and improves the efficiency of drug delivery to tumor parenchyma through the vascular barrier. Furthermore, iRGD inhibits spontaneous metastasis in mice. The antimetastatic activity is provided by neuropilin- binding RXXK but not the integrin-binding RGD motif [83]. Such peptides that have targeting properties and at the same time are able to deeply penetrate into tumor parenchyma form a separate class of peptides, CPHP (cell-penetrating homing peptides) [84].

A conjugate of iRGD with the anticancer agent abraxane (albumin-stabilized paclitaxel) is known to increase the effectiveness of abraxane and significantly reduce the overall toxicity of the drug [85, 82]. Furthermore, it was found that co-administration of the iRGD peptide with various drugs (doxorubicin, abraxane, liposomes with doxorubicin, trastuzumab) improves the effectiveness of drug penetration into tumor parenchyma and their therapeutic index [86].

Thus, short targeted peptides selected from phage libraries are increasingly being used both in diagnostic and clinical practice.

## CONCLUSION

Screening of phage peptide libraries is a fast and convenient method of obtaining organ-, tissue- and tumor- specific peptides. The safe nature of the bacteriophages for humans and simplicity of manipulations with them allowed us to obtain a wide variety of targeted peptide ligands. Some of them are in clinical trials, both as individual therapeutic agents and as vehicles for drug delivery to target organs and tissues.

The possibility of a tumor-targeted peptide application in the diagnosis and therapy of malignant neoplasms is of great interest. The small size of the targeted peptides allows them to penetrate deeply into tumor parenchyma, which is important in targeted therapy [87, 88]. Short peptides are virtually non-immunogenic, which makes them safe for clinical use [89]. Peptides can be easily modified, for example, by protection of the N- and C-termini from proteolytic degradation [87]. Chemical synthesis of short peptides is much cheaper than the production of monoclonal antibodies and recombinant proteins, while the final product does not require additional purification from bacterial cell wall components or the eukaryotic plasma membrane [90].

Tumor-specific peptides are the keys to the bulk of information about the changes that occur in cell during carcinogenesis, the mechanisms responsible for survival, proliferation, and metastasis of cancer cells. Identification of targets for such peptides is very important, but often rather not a trivial task. A tumor-specific ligand can be used for targeted delivery of diagnostic and therapeutic agents even in the absence of information on the target.
